# Toward a better understanding of the health impairment process. Types of demand and burnout component matter

**DOI:** 10.3389/fpsyt.2022.1037053

**Published:** 2023-01-09

**Authors:** Lukasz Baka, Monika Prusik, Dorota Jasielska

**Affiliations:** ^1^Central Institute for Labour Protection - National Research Institute, Warsaw, Poland; ^2^University of Warsaw, Warsaw, Poland; ^3^The Maria Grzegorzewska University, Warsaw, Poland

**Keywords:** health impairment process, challenge and hindrance stressors, job burnout, depression, human service

## Abstract

The aim of the study was to better understand the health impairment process, postulated by Job Demands-Resources (JD-R) model. Previous studies on the process have not clearly explained which types of job demands (challenge vs. hindrance) lead to depression and which burnout component (exhaustion or disengagement from work) mediates job demands—depression link. The direct and indirect (mediated *via* exhaustion and disengagement from work) effects of challenge and hindrance stressors (included 6 different demands) on depression were investigated in this 1-year cross-lagged study. Data were collected among 752 social service workers in Poland. Structural equation modeling confirmed a slightly different effects of challenge and hindrance stressors (T1) on the two components of job burnout (T2) and depression (T2). Hindrance (but not challenge) stressors were related to high depression. Hindrance stressors intensified exhaustion and disengagement from work, while challenge stressors were only associated with high exhaustion. Exhaustion (but not disengagement from work) was related to depression. These findings support the mediation function of burnout in the health impairment process but only in relation to exhaustion. They also showed that the challenge–hindrance distinction is justified also in the JD-R model. The implications for theory and research on the mental health of employees, as well as for human management practice are discussed.

## Introduction

Depression is cited as one of the main causes of low work ability, with costs borne not only by the employees, but also by the organizations and society as a whole (1, 2). Studies show that, while annually around 7% of European citizens suffer from depression (3, 4) more than 41% of the general population experience at least one episode of depression during their lifetime (5). In Poland, depression is the third cause, after cancer and pregnancy, of long-term work leaves and represents almost 8% of sick leaves for all employees per year (6). Apart from hereditary conditions (7), environmental factors, including psychosocial hazards at the workplace, e.g., job demands (8) are underlined in the etiology of depression. Job demands refer to physical, psychological, social or organizational aspects of job that require sustained physical and/or psychological effort (9). Numerous meta-analyses strongly support the important role of job demands in the development of depression (10–15), but the mechanisms for its development and the methods of reducing the risk of its occurrence remain unclear.

The Job Demand-Resources (JD-R) theory attempts to explain these mechanisms by referring to the intermediary function of job burnout, perceived as a long-term effect of chronic work-related stress caused by excessive job demands (9). In this concept, job burnout consists of exhaustion and disengagement from work. Exhaustion is a response to intensive physical, affective and cognitive strain; it manifests in fatigue, weariness and a decrease in energy. Disengagement from work is expressed by distancing oneself from work and by experiencing negative work-related affect (9). JD-R theory identified dual processes that play a role in the development of job-related strain and motivation (16). In the first of them, coined the motivational process, job resources lead to desirable organizational outcomes *via* work engagement. According to the second ones, known as the health impairment process, prolonged job demands result in depleting job resources and job burnout, which in the long run leads to depression (16). The health impairment process is the focus of the current study. Its relevance to causing depression has been confirmed in earlier studies (17–21), however, a significant number of these studies was conducted in cross-sectional (not prospective) paradigm. Moreover, in the cited studies, researchers used the aggregated job burnout indicator, without distinguishing its two components—i.e., exhaustion and disengagement from work. While many studies confirm the significant role of exhaustion (as the core of burnout) in the development of depression (22, 23), little is still known about the role of disengagement from work. In addition, much of the research on the health impairment process has been conducted in the United States and Western Europe (8, 9, 18, 24). Therefore, it seems important to conduct investigation in other countries to be able to compare results and to check whether similar mechanisms exists in different cultures.

One of the most interesting proposals of the typology of stressors in the work environment is offered by the Challenge-Hindrance Stress model (25). It posits that job stressors can be grouped into two general categories—challenge and hindrance. These two categories of stressors are theorized to exhibit differential relationships with several job-related outcomes, including performance and wellbeing. Challenge stressors refer to those demands that are perceived by the employee as an opportunity for personal development—e.g., gaining new skills, experiences, broadening horizons, strengthening self-efficacy (25). Hence, they can be a source of positive emotions and have a motivating effect. Conversely, hindrance stressors are associated with demands that conflict with other duties at work and hinder the achievement of goals and personal development. Therefore, they have a more negative impact on the mental health of the employees (25). In most previous studies on the health impairment process, this division was ignored (9). The authors somewhat arbitrarily assumed that any type of job demands (in the absence of sufficient job resources) has a negative impact on functioning of employees. This lack of distinction between these two categories of stressors some authors point out as a weakness of JD-R theory and argue that it is necessary to include this division in the future studies (9, 26). Although the Challenge-Hindrance distinction has been criticized recently (27, 28), several meta-analyses have confirmed the different effects of the two types of stressors on desirable outcomes (29–32), but mainly in the context of job performance, not mental health.

The aim of this 1-year prospective study was to test the health impairment process, including the direct and indirect (mediated *via* two components of job burnout—exhaustion and disengagement from work) effects of challenge and hindrance stressors on depression in employees involved in human service work in Poland. Many studies have documented that the risk of job burnout (and depression as its consequences) is particularly high among human service workers, whose work consists in establishing close relationships with other people based on providing help—e.g., doctors, nurses, caretakers or teachers (33, 34). These professions often attract people who are driven by a sense of mission and a desire to do good. It is indicated that the most important reasons for choosing this profession are helping others, doing interesting and challenging work, and working closely with people in need (35). Indeed, caring for others, engaging in their problems and changing their lives can be a source of positive emotions for these employees (36). The causes of job burnout are seen, among others, in exhausting relationships with other people (recipients of help), loss of faith in the effectiveness of providing help, as well as lowering professional prestige (33). Currently, working conditions in human service professions in Poland are very burdensome. According to the report *Health at a Glance Europe 2020* prepared by the OECD (37), Poland has one of the lowest numbers of employed nurses per 1,000 inhabitants in Europe (5.1 compared to an average of 8.2 for EU countries and 17.7 for the leading Norway) and doctors (2.1 compared to an average of 3.8. for EU and 6.1 for 6.1 for leading Greece). Moreover, the studies on the health impairment process have been conducted mainly in the United States and Western European countries, so it is worth testing it in an Eastern European country, where working conditions in the human service field are highly demanding (37).

### Theoretical background and hypotheses

#### Direct and indirect (mediated via exhaustion and disengagement) effects of job demands on depression

The results of studies on the direct relations between psychosocial risks at work and depression are not consistent. A significant number of cross-sectional studies shows positive connections (17, 38, 39), but the results of meta-analyses (10–15) are not equally conclusive. For example, the positive associations between quantitative demands, operationalized as the number of hours spent on work (workload), the amount of work performed (work intensity), the amount of activities performed per unit of time (time pressure) and depression were supported (8, 11). However, in the case of emotional demands—related to the need to develop emotionally close contacts with other people (e.g., patients or students)—the relationship with depression was moderate or statistically insignificant (12, 13). Particularly noteworthy are the prospective studies, with a gap of no <1 year between the first and final measurements, conducted on large sample sizes (6, 8, 12). These include national projects involving numerous cohorts of respondents, such as the Dutch NEMESIS and *Maastricht Cohort Study on Fatigue at Work*, the Belgian BELSTRESS, the English *Whitehall II*, the Danish PRISME, the French SIP *(Santé et Itinéraire Professionnel*) or the *Canadian National Population Health Survey*. In these projects, depression was diagnosed on the basis of both the subjective assessment of the participants and the opinion of specialist clinicians.

In the NEMESIS project, 2,646 employees were surveyed twice, with a 2-year interval between the assessments. High psychological demands (but not job insecurity and low job control) enabled the prediction of the occurrence of depression after 2 years (40). Similar results were obtained in the BELSTRESS project for a group of 2,821 employees with a measurement interval of more than 6½ years (41). In the French project SIP, 4,717 employees were surveyed twice at a 4-year interval, focusing on their job demands (i.e., workload, emotional demands, role conflict, job insecurity, ethical conflict, effort-reward imbalance and work-life imbalance), job resources (job control and social support) and depression (13). The results of these studies revealed that only high job insecurity and high effort-reward imbalance were associated with depression when measured after 4 years. A Canadian study using nationwide population health data from over 20,000 individuals showed *day-to-day stress* and low social support to be associated with depression, when measured after 1 year (42). In the studies cited above, researchers only tested the direct relationship between job demands and depression, disregarding potential mediators.

The JD-R model assumes that job burnout mediates the relationship between job demands and depression (9). In explaining this effect, Bakker and Demerouti (16) refer to the compensatory regulatory control model (43). According to this model, the elongation of job demands results in an increased effort of workers to maintain the required level of productivity. However, it is associated with high psychophysiological costs—activation of the sympathetic nervous system, irritability and fatigue. The prolonged high level of stressors gradually drains the employee's resources necessary to cope with stress—including time, energy, mental and physical strength, abilities, equipment and social support. This may result in job burnout and, in the long term, mental disorders. In fact, a meta-analysis of studies has shown that the strongest predictor of occupational burnout is a high level of chronic job demands (44), while prolonged burnout leads to depression (45).

Several cross-sectional (17, 21, 46, 47) and a few cross-lagged studies (18, 19) have confirmed the mediating mechanism of job burnout in the health impairment process of the JD-R framework. None of these studies, however, examined how individual job burnout components mediate the job demands—depression link. It can be assumed that exhaustion, as the core of job burnout (48), plays a key role in the development of mental disorders. Stress and long-term tension caused by physical, emotional and cognitive demands, lead to mental problems over time (49). Disengagement from work, in turn, may be a protective reaction to high job demands and in the long run prevent mental health deterioration (50). According to the Conservation of Resources [COR (51, 52)] theory, in conditions of prolonged stress, people tend to reduce their effort in the task and withdraw their commitment in order to rebuild or preserve their own resources. This can be done by slowing down the performance of work, avoiding burdens, extending breaks, as well as increasing absenteeism and delays. Such activities can serve to partially rebuild the resources invested in the coping process, as well as save and more carefully manage the rest of the resources (53). It is confirmed that employees who distance themselves from work in a stressful situation have better mental health (54). Therefore, in the current study, it is expected, that depression will be positively connected with exhaustion and negatively with disengagement from work.

#### Challenge and hindrance stressors

In the challenge—hindrance occupational stress model, typical stressors in the workplace were divided into two categories: challenge and hindrance (25). Both groups may weaken employees' wellbeing, but their impact on other job-related outcomes is usually slightly different. Challenge stressors, such as quantitative, cognitive or emotional demands, can provide the employee with benefits, in addition to their potential costs. They create opportunities for personal development, performance improvement, increase in self-efficacy, and are often a chance for a professional promotion or a salary increase. They are usually associated with a sense of fulfillment and job satisfaction, as the employee is able to overcome difficulties at work or solve difficult problems. In contrast, hindrance stressors, including role conflict, low role clarity, job insecurity or interpersonal conflicts, relate to demands that interfere in other demands and therefore they are perceived as barriers to achieving goals (25, 55). Handling them, at best, improves the employee's performance and ensures that he or she fulfills the duties imposed, but is not a source of satisfaction or fulfillment *per se* (25).

Indeed, some meta-analyses demonstrate a diverse relationship between challenge and hindrance stressors with different occupational outcomes (29–32). However, the differences relate primarily to the different impact of challenge and hindrance stressors on job performance, but not on mental health. For example, it was found that challenge stressors were positively correlated with organizational loyalty (55), work engagement (56), team performance (57), retention and organizational commitment (31) as well as with innovation (32). To the contrary, hindrance stressors were related negatively (or not related) with these outcomes. On the other hand, some studies indicate that both types of job stressors exacerbate burnout, anxiety, job strain and depression (23, 55, 58). These varied effects have been documented in numerous meta-analyzes (28–31). For example, one of them (30) found that CS and HS stressors were similarly (in terms of sign of relation) associated with strains (*β* = 0.23 and 0.50), but differently with motivation (*β* = 0.22 and −0.19), and performance (*β* = 0.21 and −0.27). In another ones (29), job burnout was related to higher levels of CS and HS (*β* = 0.10 and 0.25), however work engagement was related to higher level of CS and lower level of HS (*β* = 0.21 and −0.19). Similar regularities were observed by Podsakoff (31) with regard to strain (*β* = 0.21 and 0.48; for CS and HS, respectively), commitment (*β* = 0.29 and −0.63, for CS and HS, respectively), turnover intentions (*β* = −0.10 and 0.53, for CS and HS respectively), turnover (*β* = −0.06 and 0.25, for CS and HS, respectively), and withdrawal behaviors (*β* = −0.02 and 0.23, for CS and HS, respectively). In a more recent meta-analysis of 31 studies, Mazzola and Disselhorst (28) also showed that both HS and CS are positively related to psychological strain (ρ = 0.29 for CS and ρ = 0.36 for HS) and physical strain (ρ = 0.24 for CS and ρ = 0.38 for HS). Thus, although challenge stressors can be a source of desirable organizational outcomes, it does not mean that they are not physically, cognitively and emotionally exhausting for employees.

Moreover, a single study indicates that although challenge and hindrance stressors show a similar (positive) relationships with high exhaustion, they have differing relationships with cynicism and inefficacy (34). While the hindrance stressors intensified these undesirable outcomes, the challenge stressors showed no relation to them. If we assume, following Bakker and Demerouti (9, 16), that disengagement from work combines both cynicism (a distanced attitude toward the entire work context, i.e., people, professional duties, employee values and organizational culture) and low professional efficacy (perceiving own work as insignificant), it can be expected that this construct will be positively associated with high hindrance stressors and negatively with high challenge stressors. A certain premise for such expectations are different relations of challenge and hindrance stressors with work engagement (56), that can be perceived as opposite toward work distancing (16).

In addition, previous studies have tested a single types of hindrance and challenge stressors. Therefore, based on the typology proposed by Cavanauugh (25), two aggregated indices of stressors were created: related to hindrances and including role conflict, low role clarity and job insecurity as well as related to challenges and including quantitative, emotional and cognitive demands. Based on the cited studies, the three hypotheses were set up:

H1: Depression is positively related to both of hindrance (H1a) and challenge (H1b) stressors.
H2: Exhaustion mediates the effects of hindrance (H2a) and challenge (H2b) stressors on depression. Hindrance and challenge stressors lead to an increase in exhaustion, which in turn is related to higher depression.
H3: Disengagement from work mediates the effects of hindrance and challenge stressors on depression in different way. Hindrance stressors lead to an increase in disengagement from work (H3a), which in turn is related to lower depression. Challenge stressors lead to a decrease (H3b) in disengagement from work, which is related to lower depression.

## Materials and methods

### Participants and procedure

The study was carried out in Poland. Participants *(N* = 752) were employees belonging to three occupational groups from the social service area: (1) teachers in juvenile correctional facilities and juvenile shelters (*n* = 236), personnel of social welfare homes for the chronically mentally ill, mentally disabled children and youth (*n* = 297), and medical personnel of psychiatric and addiction treatment wards for children and youth (*n* = 219). The selection criterion for the groups listed above was that the nature of the work consisted in direct and intense contact with other people, with the aim to deliver assistance in various options: saving life and health, and constant care for the sick or individuals experiencing social problems or in conflict with the law. A systematic reviews support that human service workers are very often subjected to the wide array of work stressors, which might even cause impairment in mental health, such as burnout and depression (59, 60).

A longitudinal study was conducted, with a 1-year interval between the measurements. The study was carried out in the period between September and November 2017 (Time 1) and September–November 2018 (Time 2) by trained interviewers (i.e., employees of a company specializing in social research), at the premises of the facilities where the respondents worked. Respondents received financial rewards for participating in the research. Each participant was treated in accordance with the ethical guideline of the Helsinki Declaration. They received a hard copy of the questionnaires together with a letter explaining the aim of the study. Confidentiality of data and anonymity were provided. Participants were asked to reply to questionnaires and then to seal the questionnaires in envelopes which were then collected by the interviewers. In order to be able to match individual participants in both waves of the study, they received anonymizing identification codes. Out of 1,000 distributed questionnaires 751 (75%) returned in the first wave and 601 of them returned in the second wave of study (60% of the initial pool). They were included in the data analysis. The final sample consisted of 486 women (81%) and 115 men (18%) aged 20–77 years (M = 42.65, SD = 9.69). Work experience ranged from 1 to 47 years (*M* = 15.14, *SD* = 10.53). One-way between subjects ANOVA showed no significant differences in the distribution of age, *F*_(2, 726)_ = 2.53, *p* = 0.081, and the length of service in the three analyzed occupational groups *F*_(2, 749)_ = 1.54, *p* = 0.215. The sample size was predetermined based on requirement of at least 200 participants for any structural equation modeling (SEM) analysis as minimum (61). However, bearing in mind that our hypotheses would have required tests using models of higher complexity and of longitudinal design we decided to settle with sample of at least 500 which is more than as it is suggested by the other commonly used criteria of sample being greater 10 times than number of parameters (62).

### Measures

*Challenge and hindrance stressors* were measured with the COPSOQ II subscales (63). The aggregated indexes based on factor scores of hindrance and challenge stressors were used. The challenge stressors consisted of three COPSOQ II subscales measuring cognitive demands, emotional demands and quantitative demands. Each subscale contained four items, with answers from 1 (Always) to 5 (Never/Hardly ever). Hindrance stressors contained three COPSOQ II subscales, measuring role conflict (four items), low role clarity (three items; reversed items in order to calculate averaged index of hindrance stressors) and job insecurity (four items). Each subscale contained answers from 1 (To a very large extent) to 5 (To a very small extent). In the present study, Cronbach's alpha and McDonald's omega coefficients were: α = 0.79 and ω = 0.80 for hindrance stressors in the first measurement, and α = 0.82 and ω = 0.82 in the second measurement; α = 0.81 and ω = 0.82 for challenge stressors in the first measurement, and α = 0.82 and ω = 0.82 in the second measurement.

*Job burnout* was measured with the Oldenburg Burnout Inventory (64). The 16-item scale includes two subscales both consisting of eight items for exhaustion and disengagement from work. A five-point response scale ranged from 1 (I completely disagree) to 5 (I completely agree). The scale was characterized by good internal reliability of α = 0.77 and ω = 0.77 for exhaustion, and α = 0.77 and ω = 0.77 for disengagement from work in the first measurement, and α = 0.77 and ω = 0.78 for exhaustion and α = 0.73 and ω = 0.73 for disengagement from work in the second measurement.

*Depression* was measured with the CES-D instrument (65). The scale includes 20 items that measure how often depressive symptoms were experienced in the past week. The statements concern depressed feelings and mood, guilt and hopelessness, psychomotor downtrend and sleeping problems. A four-point response scale ranged from 0 (Rarely) or not at all (<1 day) to 3 [Most of the time or all the time (5–7 days)]. In the conducted study, Cronbach's alpha and McDonald's omega were: α = 0.91 and ω = 0.91 for the first measurement, and α = 0.92 and ω = 0.92 for the second measurement, respectively.

### Analytical procedures

At first descriptive statistics were calculated and a correlation analysis was performed. In order to determine the factor structure and to assess the parameters of fit, a confirmatory factor analysis (CFA) was carried out. In the case of aggregated indices (challenge stressors, hindrance stressors), their factor structure was also checked. The CFA was intended to confirm that the analyzed items related to job demands contain two factors: (1) challenge stressors including cognitive demands, emotional demands and quantitative demands, and (2) hindrance stressors including role conflict, low role clarity and job insecurity. Whenever it was necessary we were inspecting more closely modification indices in order to look for possible avenues of models' improvement (e.g., by introducing respecifications based on error covariances). However, the main aim was to examine the models' fit without necessity to interfere with the scale structure (e.g., removal of statements). The model fit was assessed in regard to commonly used and accepted criteria. For example, by excellent parameters we considered the values between 1.00 and 3.00 for CMIN (χ^2^/*df*), values above 0.95 for CFI, values below 0.06–0.08 for RMSEA, values <0.06–0.08 for SRMR, and above 0.05 for PClose (59). As an acceptable fit, we assumed CMIN values between 3 and 5, RMSEA values below 0.08–0.10, PClose between 0.01 and 0.05, SRMR between 0.08 and 0.10, and CFI values between 0.90 and 0.95 (61, 62, 66, 67). We did not concern, however, the significant coefficient for the χ^2^ test as an indicator of a good fit of the data as fit of the model judged based on χ^2^ test might be not adequate especially with more complex models and because of sensitivity to sample size (62, 66). After estimation of the measurement models, the internal consistency of the subscales was analyzed in a double way: using the coefficient of Cronbach's alpha but also using McDonald's omega, which is probably more adequate for the analysis of latent factors in comparison to coefficient alpha.

For the main part of the analysis, structural equation modeling was applied. Challenge stressors T1 (includes quantitative, emotional and cognitive demands, CS), hindrance stressors T1 (includes role conflict, low role clarity and job insecurity HS), job burnout T2 (JB, related to exhaustion and disengagement from work) and depression T2 were introduced into the model. The following were tested: (1) direct effects of CS/HS on depression; (2) indirect effects of JB on CS/HS—depression link.

## Results

### Confirmatory factor analysis for study variables

At first, we tested whether hindrance stressors and challenge stressors had a postulated structure. For example, we have examined whether hindrance stressors have a hierarchical structure consisting of three factors and whether the statements included in these three factors have a sufficient empirical basis to be gathered into one general index. In order to do so, we conducted a series of second-order confirmatory factor analysis (CFA). The results are presented in Table 1. Prior to the CFA analysis, all the data were investigated for violation of normality, linearity, presence and seriousness of outlying cases. No significant departures were found and we decided to continue with CFA analysis using Amos ver. 25 EN.

**Table 1:** Model adequacy and goodness of fit indices of the models tested using first- and second-order confirmatory factor analysis.

**Models**	**X^2^**	***df***	***p***	**X^2^*/df***	**RMSEA**	**PClose**	**SRMR**	**CFI**	**AIC**	***β range*** **second order**
Model—hindrance stressors	124.67	41	< 0.001	3.04	0.05	0.352	0.04	0.96	196.67	[−0.45, 0.83]
Model—hindrance stressors with modifications	93.35	40	< 0.001	2.33	0.04	0.871	0.04	0.98	167.35	[−0.46, 0.83]
Model—challenge stressors	349.99	51	< 0.001	6.86	0.09	0.000	0.06	0.88	427.99	[0.37, 1.00]
Model—challenge stressors with modifications	163.81	46	< 0.001	3.56	0.06	0.073	0.04	0.95	251.81	[0.34, 1.00]

CFA, confirmation factor analysis; RMSEA, root mean square error of approximation; PClose, p of Close Fit; SRMR, standardized root mean square residual; CFI, comparative fit index; AIC, Akaike information criterion. The respecifications of models were achieved based on error covariance modification indices.

The model fit was assessed in regard to commonly used and accepted criteria. For example, by excellent parameters we considered the values between 1.00 and 3.00 for CMIN (χ^2^/*df*), values above 0.95 for CFI, values below 0.06–0.08 for RMSEA, values <0.06–0.08 for SRMR, and above 0.05 for PClose (68). As an acceptable fit, we assumed CMIN values between 3 and 5, RMSEA values below 0.08–0.10, PClose between 0.01 and 0.05, SRMR between 0.08 and 0.10, and CFI values between 0.90 and 0.95. We did not concern, however, the non-significant coefficient for the χ^2^ test as an indicator of a good fit of the data.

The series of first- and second-order CFA results for all tested models showed excellent or acceptable parameters of model fit. Whenever it was necessary, after assessment of the basic model, some re-specifications based on error covariances were added in order to improve the fit of the models. For each analyzed model, all regression weights were significant (*p* < 0.001) and were high enough (above 0.32), according to the criteria proposed by Fidell and Tabachnick (69). Also, the intercorrelations between final second-order factors were acceptable, ranging between 0.10 and 0.52 for absolute values, which did not indicate discriminative issues. Summarizing, all assumptions about the structure of the proposed indices were confirmed. For further analysis, we have used indices based on factor scores in order to reflect the particular contribution of each item to the overall index.

### Descriptive statistics

The next step after inspection of constructs validity, was calculation of basic descriptive statistics for the study constructs (Table 2). The results of correlational analysis supported the justification of the hypothesized model. Zero-order correlations for sociodemographic characteristics are included in Appendix Table A1.

**Table 2:** Zero-order correlational coefficients for study variables separately for measurement occasions and across measurements.

	**(1)**	**(2)**	**(3)**	**(4)**	**(5)**
**Time 1**					
1. Hindrance stressors	–	0.11^**^	0.39^**^	0.41^**^	0.43^**^
2. Challenge stressors	0.11^**^	–	−0.08^*^	0.17^**^	0.08^*^
3. Exhaustion	0.41^**^	0.17^**^	**–**	65^**^	0.54^**^
4. Disengagement	0.39^**^	−0.08^*^	0.65^**^	**–**	0.42^**^
5. Depression	0.43^**^	0.08^*^	0.42^**^	0.54^**^	–
**Time 2**					
1. Hindrance stressors	–	0.05	0.31^**^	0.38^**^	0.35^**^
2. Challenge stressors	0.05	–	−0.20^**^	0.15^**^	0.08
3. Exhaustion	0.38^**^	0.15^**^	**–**	0.64^**^	0.52^**^
4. Disengagement	0.31^**^	−0.20^**^	0.64^**^	**–**	0.36^**^
5. Depression	0.35^**^	0.08	0.36^**^	0.52^**^	–
**Time 1/Time 2**					
1. Hindrance stressors	0.30^**^	**–**0.06	0.12^**^	0.14^**^	0.14^**^
2. Challenge stressors	0.05	0.30^**^	0.03	0.14^**^	0.07
3. Exhaustion	0.05	0.00	0.17^**^	0.24^**^	0.19^**^
4. Disengagement	0.06	−0.15^**^	0.25^**^	0.18^**^	0.13^**^
5. Depression	0.09^*^	**–**0.04	0.11^**^	0.13^**^	0.21^**^

* *p* < 0.05,

** *p* < 0.01,

^***^p < 0.001. Bold values indicate statistically significant.

### Main analysis—Models examination

**Figure 1:**
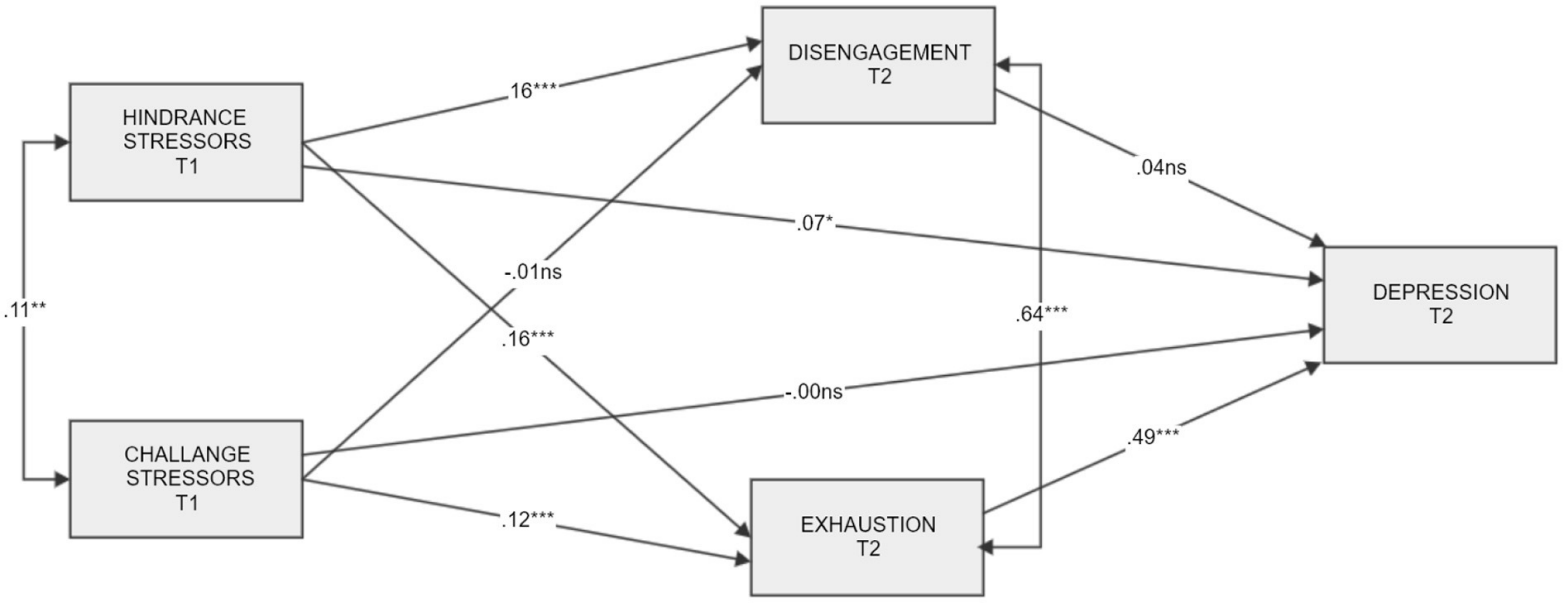
Hypothesized “just-identified” model of hindrance stressors and challenge stressors in time 1 as predictors of depression in time 2 mediated by burnout levels. Standardized coefficients are presented. “T”, time. *p < 0.05, **p < 0.01, ***p < 0.001.

**Figure 2:**
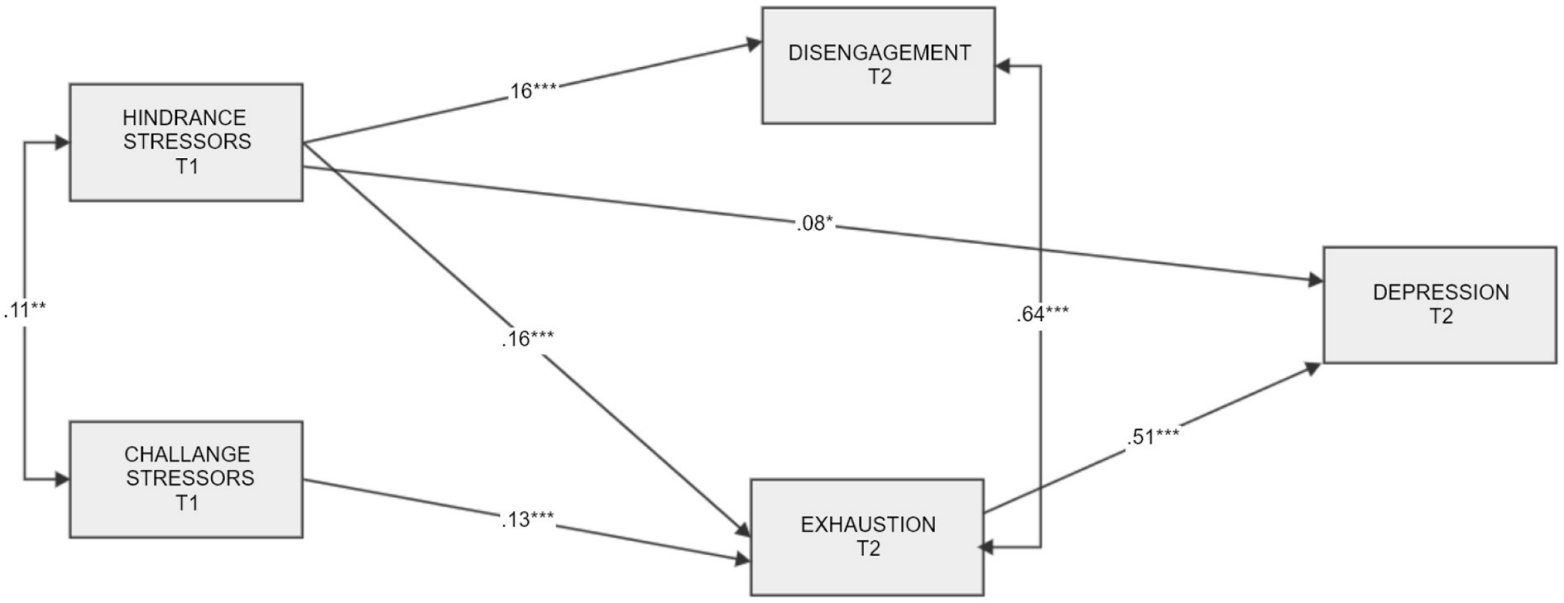
Hypothesized final trimmed model of hindrance stressors and challenge stressors in time 1 as predictors of depression in time 2 mediated by burnout levels. Standardized coefficients are presented. “T”, time. *p < 0.05, **p < 0.01, ***p < 0.001.

We have examined a hypothesized “just-identified” model by running structural equation modeling with Amos 25 enriched with additional plug-ins and estimands, for example “model fit measures” and “indirect effects” (70). Then in a step-by-step procedure we trimmed the “just-identified” model by removing three non-significant paths. According to the results obtained, the hypothesized trimmed model was characterized by excellent values of fit indices in comparison to the before mentioned criteria (please see above). The results are presented in Figures 1, 2 and Table 3.

**Table 3:** Model adequacy and goodness of fit indices of the final model.

**Models**	***X*** **^2^**	***df***	***p***	**X^2^*/df***	**RMSEA**	**PClose**	**SRMR**	**CFI**	**AIC**	**β** **range** **absolute** **value**
Hypothesized model without non-significant paths	1.23	3	0.746	0.41	0.00	0.972	0.01	1.00	35.23	[0.08, 0.51]

CFA, confirmation factor analysis; RMSEA, root mean square error of approximation; PClose, p of Close Fit; SRMR, standardized root mean square residual; CFI, comparative fit index; AIC, Akaike information criterion.

#### Hindrance stressors—Depression link via exhaustion and disengagement from work

Based on the “just-identified” model, there was a significant partial mediational effect of exhaustion in a relationship of HS and depression [this confirms H2a; Figures 1, 2), *ab* = 0.04, BCa 95% CI (0.02, 0.05), *p* = 0.001]. The mediation of HS *via* disengagement from work was not statistically significant (Figure 1 in comparison to Figure 2), *ab* = 0.003, BCa 95% CI [−0.002, 0.01], *p* = 0.318 (this does not support H3a). The total effect of HS on depression, however, was significant: higher levels of HS were related to higher levels of depression (this confirms H1a), and higher HS were also related to higher levels of disengagement from work, but there was no significant relationship between disengagement from work and depression. These results correspond with the correlational matrix (Table 2), except for the link between disengagement from work and depression, which is significant, judging by the correlational coefficient, and non-significant based on SEM analysis.

#### Challenge stressors—Depression link via exhaustion and disengagement from work

Exhaustion was a significant and a full mediator of the relationship between CS and depression, *ab* = 0.03, BCa 95% CI [0.01, 0.04], *p* = 0.002. Based on particular effects, higher levels of challenge stressors were related to higher levels of exhaustion (this supports H2b), and exhaustion was related to depression, but the total effect of CS on depression was not statistically significant (this does not confirm H1b). There was no mediational effect of disengagement from work in the CS—depression link (this does not confirm H3b), *ab* = 0.00, BCa 95% CI [−0.003, 0.001], *p* = 0.644. CS does not relate to high disengagement from work, and this, in turn, does not promote depression. Overall, the results support H1 partly and H2 partly, while H3 was not confirmed.

## Discussion

The aim of the presented 1-year prospective study was to better understand the health impairment process, postulated by JD-R model (16). In line with this process, long-termed job demands lead to an increase in job burnout and subsequent depression. Although the mediational effect of burnout has been already confirmed in previous studies (18, 19), only the aggregate job burnout index has been used in them, without division for two of its main components—exhaustion and disengagement from work. While the role of exhaustion in the development of depression is strongly documented (22, 23), the results of research on disengagement from work are ambiguous. Some of them show that it leads to undesirable outcomes (71), others that it may have a protective function and reduces the job strain (50, 53). The previous studies on the health impairment process have not categorized also job demands into challenge and hindrance (9, 26). In addition, most of them tested the relationships between demands, burnout and depression in the cross-sectional studies paradigm. Therefore, we investigated, what type of job demands (related to challenge or hindrance) and which component of job burnout (exhaustion and disengagement from work) affects the development of depressive symptoms, after 1 year.

We expected that both HS and CS would be positively related to high depression directly and indirectly *via* exhaustion and disengagement from work, but in different way. Specifically, while HS would lead to higher exhaustion and higher disengagement from work, CS would be related to higher exhaustion and lower disengagement. Depression, in turn will be associated with high exhaustion and low disengagement from work. The obtained results supported both the total effect of HS on depression with higher levels of HS related to higher levels of depression and different functions of the two job burnout components. We found that exhaustion (contrary to disengagement) mediates the undesirable impact of HS and CS on depressive symptoms. Although HS led to high disengagement from work, there was no relationship between disengagement and depression. These findings support health impairment process, postulated by JD-R model (16) and documented in previous studies (18, 19), but only for one job component of burnout. Professionals who are exposed to prolonged stress tend to deplete their own coping resources over time, leading to exhaustion and, in the long term to depression (16, 23).

The observed lack of relationship between disengagement from work and depression requires a more detailed discussion, as it is responsible for the failure to confirm H3. Research findings on this topic are quite confounded. On the one hand, some studies show that both depersonalization/cynicism (e.g., labeling, using professional jargon, reducing commitment) and withdrawal (lowering effort, doing work more slowly, deliberately being late, shortening work time, extending breaks, illness simulation) may be ways to conserve or rebuild stress-depleted job resources (50, 51) and thus help in more effective dealing with excessive job demands and negative emotions at work (52, 53). Such strategic resource management is a prerequisite for 'good' health. For example, a study has confirmed that distancing from work can be helpful in maintaining mental health under high workload conditions (53). On the other hand, some studies showed that distancing from work and avoiding or postponing unpleasant job tasks or uncomfortable situations at workplace does not solve those problems that are a source of constraints and tension. The tasks that a worker avoids do not disappear. Instead, the workload accumulates and the employee is constantly worried about delayed tasks, which drains his/her energy and can be a source of frustration (72). Moreover, by avoiding demanding professional tasks and postponing them, the employee reduces his own chances of acquiring new skills, weakens the sense of control over work (73), which may result in deterioration of mental health. Finally, there are indications that the less an employee is involved at work, the less they will be worried when circumstances are far from perfect. According to the career orientation concept (71), when current work does not provide satisfaction or fulfillment, it can be perceived as a source of salary only (job orientation) leading to minimal involvement. Therefore, disengagement from work does not have to lead to depression. In fact, it may be a form of adjustment to an adverse situation.

The obtained research results only partially confirmed the different effects of HS and CS on the two components of job burnout. As expected, employees who experienced the high level of HS felt more exhausted and less engaged with the job after a year. However, it turned out that CS also leads to negative outcomes, admittedly only in relation to exhaustion (but not to disengagement from work). It can therefore be concluded that both stressors are job characteristics which in long run consume resources and therefore may be the source of exhaustion, however, CS other than HS also allow for potential resource gains, personal development and may keep engagement (73). The obtained results are largely consistent with the challenge—hindrance occupational stress model (25). According to it, when job demands create opportunities for the employee toward personal development, acquiring new skills or strengthening self-efficacy, it seems that a more frequent response to them is an increase in commitment rather than a decrease in it (30, 31). It does not mean, however that they are not cognitively and emotionally aggravating (57). The different relationship patterns CS and HS with motivational and health outcomes have been demonstrated in several the meta-analytical studies (29–32). The results of our research are in line with them. They showed that while both HS and CS may deplete employee resources, they affect employee engagement in a different way. Therefore, challenge—hindrance distinction may be important in the prediction of health and motivational outcomes.

### Limitations and future research

The authors of the presented research are aware of some of its limitations. Although, on the basis of the results of prospective studies with double measurement of variables, one can conclude about the order of occurrence of the studied phenomena (and thus the direction of the relationship between the studied variables), it is not justified to draw conclusions about the cause-effect relationships (74). This requires more in-depth analyzes of cross-lagged effects or experimental studies. However, manipulating the level of job demands and/or job resources would be difficult to implement and would raise serious ethical problems. Another limitation is the number of measurements. In this two-wave study, burnout and depression were tested at the same (the second) point of measurement. The optimal solution would be to conduct a three-wave study with a separate measurement of mediational and dependent variables (19). Regarding the generalization of the results, it should be remembered that quite specific professional groups participated in the research—teachers in juvenile correctional facilities and juvenile shelters, personnel of social welfare homes for the mentally disabled children and youth as well as medical personnel of psychiatric and addiction treatment wards for children and youth. Working in these professions requires entering into very close and often very emotionally burdensome relationships with children and adolescents. Therefore, it is not legitimate to draw any conclusions on a wider working population. The observed regularities concern a very narrow segment of social service workers and should not be generalized to other market sectors. The last issue is the gender imbalance in the research sample. Women dominated because the number of women in the analyzed professions was significantly higher. For the male population, in traditionally male occupations, the results would probably be different.

A polemical issue may be the arbitrariness of classifying stressors as challenge and hindrance, in our study. While building the aggregated indicators of challenge and hindrance stressors, we were guided by theoretical premises (25), however, several studies confirm that several variables, including individual differences (75), appraisals (76), access to high job resources (77) and the level of professional efficacy (78) and competences (27), play a great role in classifying a given stressor to the challenge or hindrance group. Thus, individuals can appraise stressors differently, based on any number of internal and external variables. Moreover, the perception of stressors shows quite high intra-person variability. This means that 1 day employees can be very positive about their work, and the next day that positive attitude can change. So it is quite possible that an employee may perceive some of their job tasks as challenges on 1 day and feel that the same tasks are limiting them on another day (27). Perhaps not without significance is also the fact that the studied sample was dominated by women who, as shown by the results of the research (28), experience a lower level of challenge stressors. Some researchers emphasize that when assessing stressors as challenge and hindrance, their stability over time is also important (79). For example, when challenge stressors were stable week by week, workers were able to predict them better compared to periods of high fluctuation of these stressors. As a result of predicting stressors, people found them difficult and eventually experienced less overall stress. Those who experienced greater fluctuation in challenge stressors and rated them as more restrictive showed lower performance and experienced lower wellbeing due to lower stressor prediction (79).

In future studies, it would be worth investigating the role of personal and job resources. The authors of the JD-R model strongly emphasize the role of job resources as factors that may reduce job demands (and the associated physiological and psychological cost), are functional in achieving work goals, and stimulate personal growth, learning and development (9). Therefore, it would be worth checking whether different kinds of job resources (e.g., social support or job control) moderates differently for challenge vs. hindrance demands. In relation to personal resources, two studies have shown that conscientiousness can moderate the impact of CS and HS on job performance in a different way (80, 81). While in the case of CS, the highest level of job performance is achieved by the most conscientious employees, in the case of HS it is exactly the opposite. It would be worth including other types of personal resources (e.g., optimism, self-efficacy) in future studies. Particularly useful would be those concerning specific personal resources related to a professional context (e.g., occupational hardiness or occupational resilience). As suggested by some authors, it is specific resources related to the professional context that are particularly effective in reducing stress (82). The question of whether and how way different interactions of job and personal resources moderates challenge and hindrance demands is also open, and it is ripe for investigation.

### Practical implications

Our results yield some practical implications for fostering employee's wellbeing in organization. With growing number of empirical evidence about benefits from investing in workers' happiness (83) it seems particularly important to identify factors that affect this state. The present study has shown that high level of HS may have adverse effects for subjective wellbeing on many dimensions. Therefore, any organization, that aims to create a people oriented workplace should scrutinize their impact on employees. HS can arise from any factors that impede goal accomplishment, such as organizational politics, excessive bureaucracy, ambiguous job demands, job insecurity (23). They can also operate on interpersonal level—for example conflicts hinder effective communication which is often essential for good job performance. By recognizing and managing HS organization can offer a substantial support for the workers and, consequently, lower the risk of depression and both elements of job burnout—disengagement and exhaustion. Yet, practical implications are not limited to recommendations for management. Employees at every level can make an effort to diminish the level of HS by using job crafting at work. This term refers to proactive behaviors performed by employees that lead to changing tasks and interactions at work in order to improve job satisfaction (such as the deliberate use of time management techniques or delegating). Research shows that by using job crafting, employees can modify the level of their job demands and resources (84). Our study indicates that this can be a meaningful strategy for lowering the risk of depression.

Some practical remarks can also be made regarding challenge stressors, although they were not related to the level of depressive symptoms and to disengagement, the were associated with exhaustion. This implies that as long as the work requires high cognitive and emotional engagement it can potentially lead to depletion of resources and can be harmful to wellbeing (16, 23). Therefore, implementing strategies aimed at keeping work-life balance and having effective rest seem to be essential for maintaining the long term job satisfaction, irrespective of the work engagement.

## Conclusion

This 1-year prospective study has confirmed in part the health impairment process, postulated by JD-R and justified the distinction of stressors into challenge and hindrance. It found that the two groups of stressors had different impact both on depression and job burnout. Hindrance stressors (but not challenge stressors) had the direct impact on depression. This direct effect was mediated by job burnout, but only in relation to exhaustion (not disengagement from work). Disengagement from work was not associated with depression. Although both types of stressors were related to higher exhaustion, only HS was related to disengagement from work. These findings are consistent with job stress models and some results of meta-analytical studies, in general.

## Data availability statement

The raw data supporting the conclusions of this article will be made available by the authors, without undue reservation.

## Ethics statement

Ethical review and approval was not required for the study on human participants in accordance with the local legislation and institutional requirements. The patients/participants provided their written informed consent to participate in this study.

## Author contributions

LB: conceptualization, methodology, investigation, resources, writing—original draft, supervision, and project administration. MP: methodology, investigation, formal analysis, resources, and writing—original draft. DJ: methodology, investigation, data curation, writing—original draft, and visualization. All authors contributed to the article and approved the submitted version.

## Appendix

**Table A1:** Zero-order correlational coefficients for study variables and sociodemographic characteristics separately for measurement occasions.

	**Gender, 1-F**	**Age**	**Education^a^**	**Seniority at work**	**Seniority at work, sqrt^a^**
**Time 1**					
1. Hindrance stressors	–0.02	**–0.14^**^**	–0.05	–0.05	**0.09^*^**
2. Challenge stressors	**0.10^**^**	–0.01	**0.26^**^**	0.04	0.01
3. Disengagement	–0.02	–0.07	**–0.21^**^**	–0.03	0.02
4. Exhaustion	0.05	0.07	**–0.12^**^**	**0.12^**^**	0.05
5. Depression	0.02	0.03	**–0.10^**^**	0.06	0.04
**Time 2**					
1. Hindrance stressors	–0.03	**–0.10^*^**	0.00	–0.06	0.01
2. Challenge stressors	**0.08^*^**	–0.04	**0.19^**^**	0.02	–0.05
3. Disengagement	**–0.08^*^**	–0.06	**–0.10^*^**	–0.03	0.02
4. Exhaustion	0.03	–0.04	–0.07	–0.02	–0.03
5. Depression	0.04	0.01	**–0.10^*^**	0.05	0.01

* *p* < 0.05,

** *p* < 0.01,

^***^*p* < 0.001.

a Education was slightly negatively skewed but it did not require transformation, while seniority at work was slightly positively skewed and it did require sqrt transformation. Bold values indicate statistically significant.
